# Role of *Campylobacter jejuni* gamma-glutamyl transpeptidase on epithelial cell apoptosis and lymphocyte proliferation

**DOI:** 10.1186/1757-4749-6-20

**Published:** 2014-06-12

**Authors:** Pauline Floch, Vincent Pey, Michel Castroviejo, Jean William Dupuy, Marc Bonneu, Anaïs Hocès de la Guardia, Vincent Pitard, Francis Mégraud, Philippe Lehours

**Affiliations:** 1 Bacteriology Laboratory, University of Bordeaux, F-33000 Bordeaux, France; 2 INSERM U853, F-33000 Bordeaux, France; 3 MCMP, UMR 5234, CNRS, University of Bordeaux, F-33000 Bordeaux, France; 4 Centre de Génomique Fonctionnelle, Plateforme Protéome, University of Bordeaux, F-33000 Bordeaux, France; 5 CNRS, UMR 5164, CIRID, University of Bordeaux, F-33000 Bordeaux, France; 6 INSERM U853, Bacteriology Laboratory, Université de Bordeaux (site Carreire), F-33076 Bordeaux, France

**Keywords:** *Campylobacter jejuni*, GGT, Lymphocytes, Epithelial cells

## Abstract

**Background:**

A gamma-glutamyl transpeptidase (GGT) is produced by up to 31% of strains of *Campylobacter jejuni* isolates. *C. jejuni* GGT is close to *Helicobacter pylori* GGT suggesting a conserved activity but unlike the latter, *C. jejuni* GGT has not been studied extensively. In line with the data available for *H. pylori*, our objectives were to purify *C. jejuni* GGT from the bacteria, and to evaluate its inhibitory and proapoptotic activities on epithelial cells and human lymphocytes.

**Methods:**

*C. jejuni* GGT was purified from culture supernatants by chromatography. After verification of the purity by using mass spectrometry of the purified enzyme, its action on two epithelial cell lines and human lymphocytes was investigated. Cell culture as well as flow cytometry experiments were developed for these purposes.

**Results:**

This study demonstrated that *C. jejuni* GGT is related to Helicobacter GGTs and inhibits the proliferation of epithelial cells with no proapoptotic activity. *C. jejuni* GGT also inhibits lymphocyte proliferation by causing a cell cycle arrest in the G0/G1 phase. These effects are abolished in the presence of a specific pharmacological inhibitor of GGT.

**Conclusion:**

*C. jejuni* GGT activity is comparable to that of other Epsilonproteobacteria GGTs and more generally to *Helicobacter bilis* (inhibition of epithelial cell and lymphocyte proliferation, however with no proapoptotic activity). It could therefore be considered as a pathogenicity factor and promote, via the inhibition of lymphocyte proliferation, the persistence of the bacteria in the host. These observations are consistent with a role of this enzyme in the pathophysiology of chronic infections associated with *C. jejuni*.

## Background

*Campylobacter jejuni* is the main *Campylobacter* species isolated from humans. The *C. jejuni* reservoir consists essentially of the digestive tract of birds, including poultry. Transmission to humans occurs mainly indirectly via food or contaminated water [1]. In humans, Campylobacters induce enteritis generally with a favourable evolution after a few days but with potential complications like systemic infections or post-infectious diseases (Guillain–Barré syndrome). *C. jejuni* may also be involved in immunoproliferative small intestinal disease (IPSID) [2] which belongs to the group of digestive mucosa associated lymphoid tissue (MALT) lymphomas.

In *Helicobacter pylori*, a bacterium close to *C. jejuni*, the role of gamma-glutamyl transpeptidase (GGT) has been extensively studied [3-5]. GGT is an enzyme belonging to the family of N-terminal nucleophile hydrolases, present in prokaryotes and eukaryotes, playing a major role in the degradation of glutathione. GGTs of distant species (mammals and bacteria) often exhibit a high protein sequence identity (>25%) [6,7].

*H. pylori* GGT has been the subject of numerous studies. It is present in 100% of the strains and is constitutively expressed. It can reach the periplasmic space thanks to its signal peptide. It is synthesized as an inactive 60 kDa precursor which undergoes autocatalytic cleavage resulting in an active heterodimer composed of two subunits of 20 and 40 kDa respectively. The small subunit plays a key role in the autocatalytic and enzymatic activity of GGT; it includes the active site of the enzyme [6,8]. This enzyme is not essential to the bacteria, as *ggt* gene deletion does not inhibit bacterial growth, but it provides an advantage in gastric colonization [3]. *H. pylori* GGT also plays a role in the inhibition of T-lymphocyte proliferation by blocking the cell cycle in the G1 phase [5]. In addition, several studies have shown that *H. pylori* GGT has a proapoptotic effect on human gastric epithelial cells (AGS line) [9-11].

*C. jejuni* GGT has been studied less. It is present in up to 31% of strains [12] and has 67 to 69% of amino-acid identity with *H. pylori* GGT. The cleavage site, the essential residues for enzymatic activity, substrate recognition and catalytic activity for *H. pylori* GGT are conserved in *C. jejuni* GGT. It allows *C. jejuni* to metabolize glutamine and glutathione as a source of amino acids and possibly to persist in the intestine [13]. A Finnish study showed that *C. jejuni* GGT could be a marker of severity of infection, in particular for bloody diarrhea [14].

In this study, we used phylogenetic and functional approaches to analyze *C. jejuni* GGT. We showed that *C. jejuni* GGT is related phylogenetically to Helicobacter GGTs and, like *H. pylori* GGT, *C. jejuni* GGT inhibits lymphocyte and epithelial cell proliferation. The inhibition observed was mediated by an apoptosis-independent mechanism, suggesting a conserved function among GGTs in Epsilonproteobacteria.

## Results

### Phylogenetic analysis

The phylogenetic position of *C. jejuni* GGT among Epsilonproteobacteria was analyzed (Additional file 1: Figure S1). *C. jejuni* GGT was closer to *H. bilis*, *Helicobacter canis* and *Helicobacter trogontum* GGTs than to *H. pylori* GGTs. *C. jejuni* GGTs appeared to be highly conserved, including those of *H. pylori.*

### *C. jejuni* GGT purification

**Figure 1:**
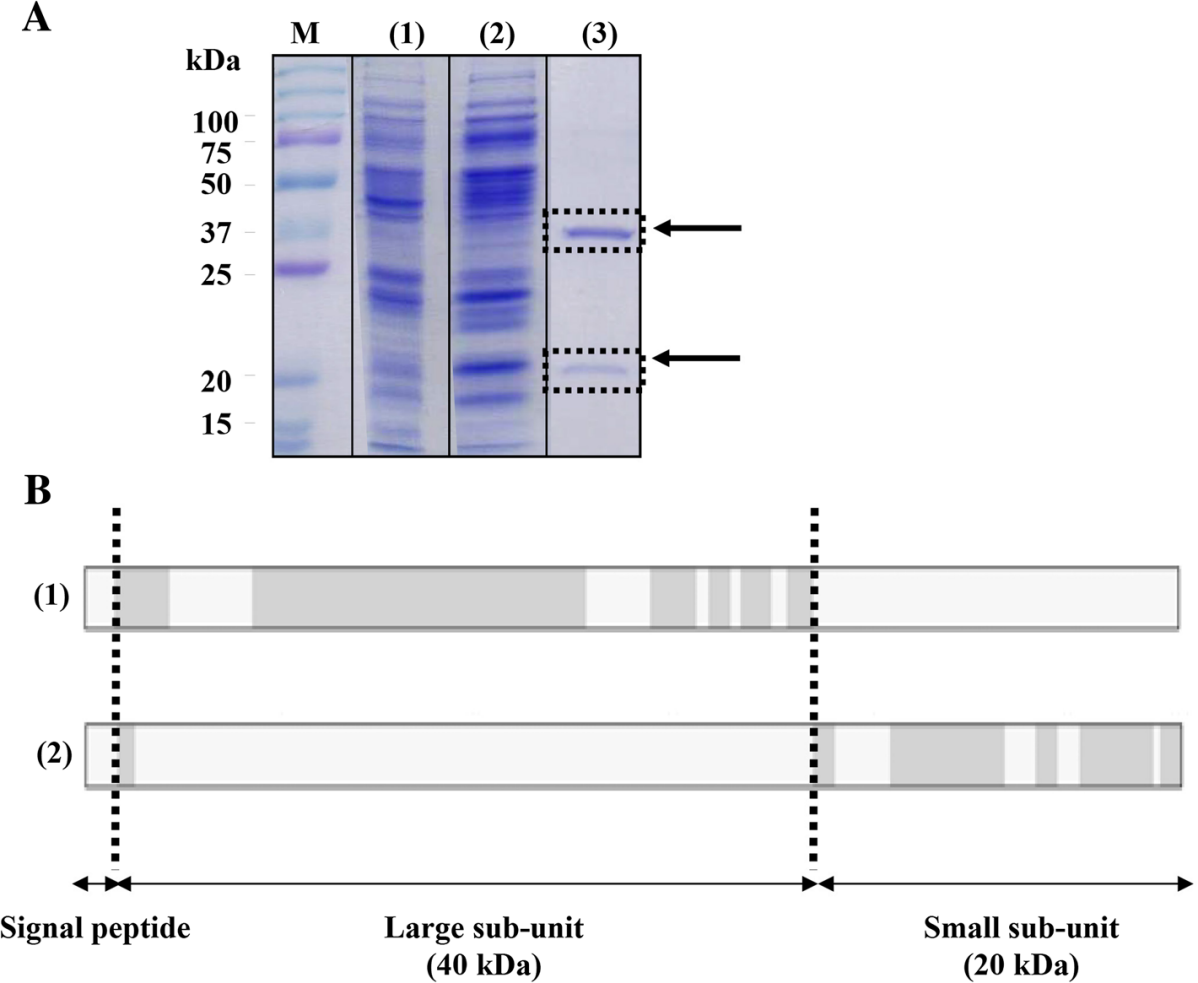
***C. jejuni *GGT purification. (A)** Analysis of the purification of *C. jejuni* GGT by migration on SDS-PAGE gel and Coomassie blue staining. M: size marker. (1) Supernatant after precipitation and dialysis, (2) After the first ion exchange chromatography, (3) Final elution. The black arrows indicate the 40 and 20 kDa bands which correspond to the expected molecular weights of the large and small subunits of *C. jejuni* GGT, respectively. The black dotted boxes represent the gel bands which were cut and analyzed by mass spectrometry. **(B)** Mass spectrometry results. After extraction and protein digestion, (1) amino-acids found in the 40 kDa band (in grey) cover 73.6% of the protein sequence of the large subunit of *C. jejuni* GGT; (2) amino-acids found in the 20 kDa band cover 68.8% of the protein sequence of the small subunit.

*C. jejuni* GGT was purified from a bacterial supernatant. Briefly, as described in Materials and Methods, proteins from a supernatant were first precipitated with ammonium sulfate. The supernatant was then dialyzed and purified by two ion-exchange chromatographies. To determine the effectiveness of the purification, the dialysate, the product obtained after the first chromatography and the final product were analyzed by migration on a SDS-PAGE gel and Coomassie blue staining (Figure 1A). Efficient purification was observed between the dialysate, the product of the first chromatography and the final product.

Two bands at approximately 40 and 20 kDa were observed on the gel after the final purification, which is consistent with the expected molecular weights of the large and small subunit of *C. jejuni* GGT, respectively. These bands were cut and analyzed by mass spectrometry (after extraction and protein digestion). The results showed the presence of *C. jejuni* GGT with a significant number of peptides (Figure 1B): the amino-acids found in the 40 kDa band represent 73.6% of the protein sequence of the large subunit of *C. jejuni* GGT and those found in the 20 kDa band represent 68.8% of the protein sequence of the small subunit.

### *C. jejuni* GGT effects on epithelial cells

**Figure 2:**
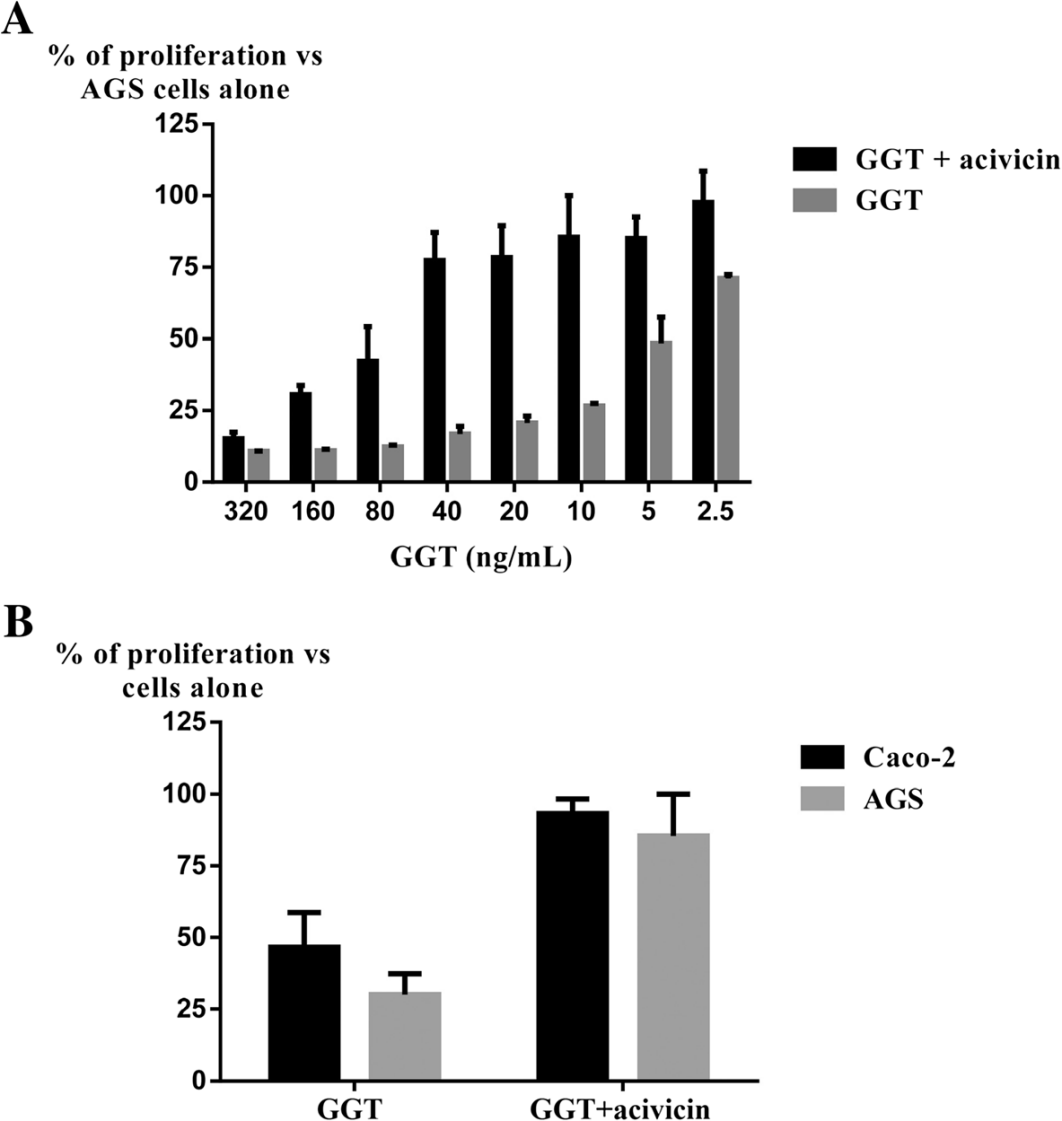
**Inhibitory effect on epithelial cells proliferation by *C. jejuni *GGT. (A)** AGS cells were cultured for 24 h with *C. jejuni* purified GGT at different concentrations (from 320 to 2.5 ng/mL) preincubated or not with acivicin (10 μM). **(B)** Caco-2 cells in parallel with AGS cells were cultured for 24 h with *C. jejuni* purified GGT at 10 ng/mL, preincubated or not with acivicin (10 μM). For each experiment, the percentage of growth proliferation is calculated relative to the proliferation of cells in their standard culture medium whithout GGT. The data are consistent with the results obtained during one experiment performed in triplicate, representative of the results for three independent manipulations.

The gastric epithelial cell line AGS was cultured for 24 h in media supplemented with 2.5 to 320 ng/mL of purified GGT with or without acivicin to determine the optimal concentration to evaluate GGT activity. At high concentrations epithelial cell proliferation was strongly inhibited with or without GGT inactivation with acivicin suggesting an artifactual phenomenon. This artifactual effect was lost at lower concentrations (Figure 2A). At 10 ng/mL of GGT, a significant reduction of AGS cell proliferation was still observed whereas acivicin restored a normal proliferation rate. This concentration was finally chosen as the lowest GGT concentration to be used. The activity of GGT at 10 ng/mL was also verified on Caco-2 cell proliferation (Figure 2B). Compared to AGS cells, GGT also had a significant effect on intestinal cell proliferation.

Trypan blue staining and cell counts after a 24 h incubation with *C. jejuni* GGT was used to verify that this inhibitory effect was not due to a cell death phenomenon, rather to a cell proliferation arrest (data not shown).

**Figure 3:**
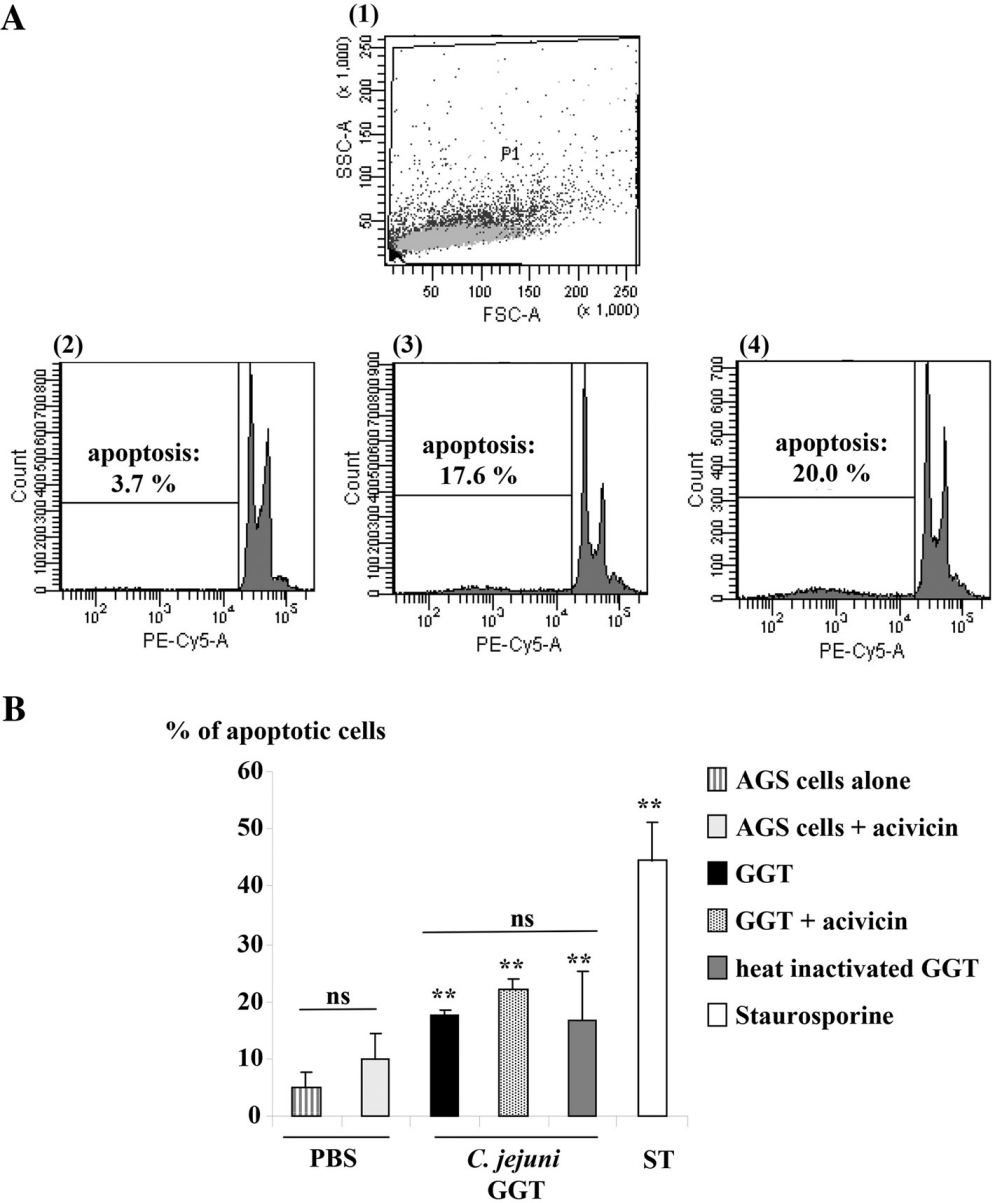
**Activity of *C. jejuni *GGT on AGS cells apoptosis. (A)** 1 - Selection of the population of interest (P1) depending on the size (FSC-A) and cell density (SSC-A). The population was chosen as large as possible in order to properly select the apoptotic cells that may have varied cytological features. Fluorescence emitted at cyanine 5 wavelength (PE-Cy-5) was analyzed based on the number of events in the selected population: diploid cells are separated from hypodiploid cells (apoptotic cells). The proportion of epithelial cells in apoptosis was determined by the ratio between hypodiploid cells and the number of events in the P1 population. Typical cytometry results are shown for 2- control AGS cells, 3- AGS cells with *C. jejuni GGT* (10 ng/mL) and 4- AGS cells with *C. jejuni* GGT (10 ng/mL) preincubated with acivicin (10 μM). **(B)** AGS cells were cultured for 24 h with *C. jejuni* GGT (10 ng/mL) and preincubated or not with acivicin (10 μM). A significant difference was observed between control cells and the cells with *C. jejuni* GGT. However, preincubation for 2 h at 37°C with acivicin or heat inactivation (70°C, 20 min) of *C. jejuni* GGT had no effect. Staurosporine (10 μM) was used as the positive control. These data presented in **A** and **B**, are consistent with the results obtained during the same manipulation carried out in triplicate, representative of the results for three independent manipulations. (**indicates a significant difference, p <0.05 versus cells in PBS alone; ns for a non-significant difference, p > 0.05).

The activity of *C. jejuni* GGT on apoptosis was then studied by flow cytometry on AGS cells only. Compared to the control, *C. jejuni* GGT induced a significant increase in the percentage of apoptosis (Figure 3A). However, *C. jejuni* GGT preincubation with acivicin did not decrease the percentage of epithelial cells undergoing apoptosis. In conclusion, the proapoptotic activity observed in the presence of *C. jejuni* GGT did not seem to be dependent on the presence of GGT (Figure 3B) but on contaminant proteins even when they were in very small amounts in the final product.

### *C. jejuni* GGT effects on human lymphocytes

**Figure 4:**
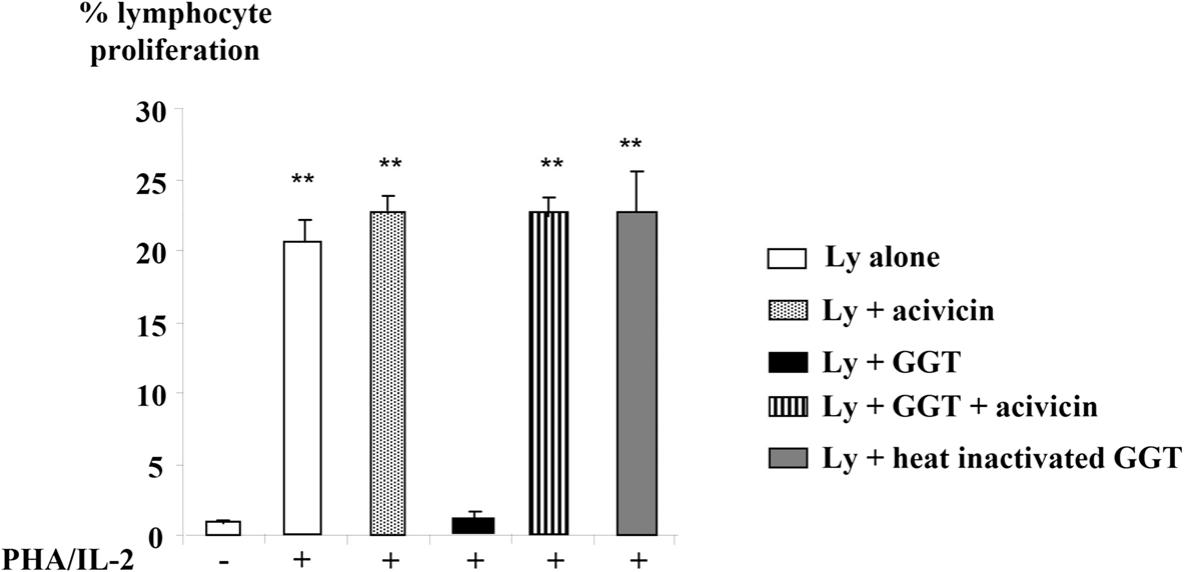
**Inhibitory effect on lymphocyte proliferation of *C. jejuni *GGT.** Lymphocytes were cultured with *C. jejuni* purified GGT (10 ng/mL). The ability of lymphocyte proliferation was verified by the action of phytohemagglutinin (PHA, 1 mg/mL) associated with interleukin-2 (IL-2, 20 U/mL). Proliferation was measured after 4 days of culture by BrdU incorporation. Preincubation with acivicin (10 μM) or the prior inactivation by heat (20 min, 70°C) of *C. jejuni* GGT restored lymphocyte proliferation. These data are consistent with the results obtained during a manipulation performed in triplicate and representative of the results for three independent manipulations. (**indicates a significant difference, p <0.05, compared to lymphocytes alone (Ly)).

Lymphocyte proliferation was measured after 4 days of culture in the presence of. *C. jejuni* GGT. A significant inhibition of lymphocyte proliferation was observed. Preincubation of *C. jejuni* GGT with acivicin or heat inactivation of the enzyme, restored a level of lymphocyte proliferation similar to that of the lymphocytes alone (Figure 4). The inhibition of lymphocyte proliferation could therefore be attributed to the GGT.

No significant apoptosis was observed in the presence of *C. jejuni* GGT. The lymphocyte cell cycle was however disturbed and, in particular, a cell cycle arrest in the G0/G1 phase was found (Table 1) (Additional file 2: Figure S2). No disturbance was observed when *C. jejuni* GGT was inactivated with acivicin or heating.

**Table 1:** **Distribution of cell cycle phases in lymphocytes cultured with *C. jejuni *** **GGT**

	**Cell cycle phase**		
	**G0/1**	**S**	**G2/M**
**Ly**	99.0 +/- 0.2	0.6 +/- 0.2	0.2 +/- 0.1
**Ly + PHA/IL-2**	69.9 +/- 0.8	18.0 +/- 0.7	11.1 +/- 1.3
**Ly + PHA/IL-2 + acivicin**	71.3 +/- 2.0	18.0 +/- 0.7	8.0 +/- 2.1
**Ly + PHA/IL-2+ *C. jejuni *** **GGT**	97.5 +/- 0.6	1.2 +/- 0.5	1.0 +/- 0.1
**Ly + PHA/IL-2+ *C. jejuni *** **GGT + acivicin**	68.6 +/- 1.9	17.7 +/- 0.9	12.3 +/- 0.7
**Ly + PHA/IL-2+ heat inactivated *C. jejuni *** **GGT**	71.5 +/- 1.0	16.8 +/- 2.0	9.6 +/- 0.8

Flow cytometry study by Nicoletti assay after 4 days of culture with *C. jejuni* GGT (10 ng/mL). Percentage of cells in G0/G1, S and G2/M phase depending on growth conditions. These data are consistent with the results obtained during a manipulation performed in triplicate representative of the results for two independent manipulations. (Ly: lymphocytes, PHA: phyto hemagglutinin, IL-2: interleukin 2).

In conclusion, *C. jejuni* GGT exhibited significant biological effects on human lymphocytes.

## Discussion

Because of the conserved protein homology of *C. jejuni* GGT with *H. pylori* GGT, the objectives of the present study were to determine whether *C. jejuni* GGT had the same properties as *H. pylori* GGT to inhibit 1) epithelial cell proliferation via a pro-apoptotic mechanism and 2) human lymphocyte proliferation. These data could be strong arguments to better understand the pathogenicity of *C. jejuni* and to consider *C. jejuni* GGT as an important pathogenicity factor of this bacterium as well.

*C. jejuni* GGT was highly conserved among the *C. jejuni* strains. As already shown by Skarp-de Haan CP *et al.*, [15]*C. jejuni* GGT was also very close to *Helicobacter* GGTs, in particular those from *H. bilis*, *H. canis* and *H. trogontum*, which suggests a conserved activity between these GGTs. *C. jejuni* GGT was purified directly from culture supernatants. This technical approach allowed the isolation of the protein directly from the bacterium, avoiding any potential problems of protein solubility or refolding as described in a previous publication [7].

In line with results concerning *H. pylori*, *H. bilis*, and *H. suis* GGTs [7,10,16,17], an inhibition of epithelial cell proliferation was observed for *C. jejuni* GGT. This biological activity seemed to be independent of the cell origin (gastric or intestinal). It has been suggested that *H. pylori* and *H. suis* GGTs exert their inhibitory activity indirectly via the formation of metabolites during transpeptidation [5,16]. This point should be investigated further for *C. jejuni* GGT. Contrary to what has been described for *H. pylori* and *H. suis* GGTs [9-11,16], this inhibitory effect does not depend on a proapoptotic activity of *C. jejuni* GGT. *H. pylori* and *H. suis* GGTs are indeed responsible for the apoptosis of human gastric epithelial cells (AGS cell line), via the activation of caspases 3 and 9, Bax, a decreased expression of anti-apoptotic proteins Bcl-2 and Bcl-xl, and the release of cytochrome c [10,11] or via the increase in H_2_O_2_ concentration due to glutathione catabolism by GGT [16,18]. A necrotic phenomenon was also described for *H. suis* GGT. In the study of Shibayama *et al.* fetal calf serum (FCS) deprivation in the culture medium was carried out before the apoptosis study [9]. Therefore if the proapoptotic activity is detectable only under stress conditions, it may simply be a technical artifact. Finally, our results are similar to those of Rossi *et al.* with *H. bilis*[7], which interestingly is one of most closely related phylogenetically to *C. jejuni* GGT [15].

The role of *C. jejuni* GGT in the inhibition of lymphocyte proliferation with cell cycle arrest in the G0/G1 phase was also demonstrated in our study. This property is shared by *H. pylori*, *H. bilis* and *H. suis* GGTs [4,5,7,19,20]. As for *H. pylori* GGT, the inhibition of lymphocyte proliferation is not dependent on an apoptotic phenomenon. Schmees *et al.* showed that *H. pylori* GGT acts on the cell cycle via a decrease in cellular levels of Ras-dependent track mediators [5]. The cell cycle arrest in the G1 phase by *H. pylori* GGT is characterized by an increase in p27 and CDK inhibitor levels and a decrease in cyclins. As for its activity on epithelial cells, GGT appears to have an indirect action via the metabolites formed during transpeptidation [20]. These mechanisms have to be validated for *C. jejuni* GGT.

## Conclusions

*C. jejuni* GGT promotes the persistence of intestinal colonization in animals [21] and in our study it inhibited the proliferation of human epithelial cells and lymphocytes. These inhibitory activities are shared by *H. pylori* GGT [4,5,19]. *C. jejuni* GGT could therefore be considered as a pathogenicity factor for this bacterium, fostering the persistence of the infection in humans via the inhibition of lymphocyte proliferation.

## Methods

### Bacterial strains, cell lines, culture conditions

Eleven *C. jejuni* strains named 2003–198, 2002–200, 2004–304, 2003–383, 2004–438, 2002–608, 2002–646, 2007–741, 2003–795, 2003–1129, and 2003–1206 were obtained from the National Reference Center on Campylobacters and Helicobacters (CNRCH) strain collection (Univ. Bordeaux, Bordeaux, France). They were received between 2002 and 2007. All of these strains were isolated from humans, essentially from faecal samples, and they were all identified at the species level by MALDI-TOF mass spectrometry [22]. They belong to Lior biotype III (hippurate +, H_2_S + and DNase -), in which the prevalence of GGT is more important (60% according to personal data) than in the other biotypes (15-20%). They were used to assess the diversity of GGT genetics in *C. jejuni. C. jejuni* strain 81116 (a kind gift from Dr D. Newell) was used to purify GGT and to study *C. jejuni* GGT activity on epithelial cells and lymphocytes. It was selected for its GGT activity, as previously described by Barnes *et al.*[21].

All strains were grown on horse blood agar (bioMérieux, Marcy l’Etoile, France) in a microaerobic atmosphere at 37°C. A microscopic control was systematically performed to check the morphology and mobility and standard bacteriological tests (Gram staining, catalase, oxidase) were carried out as well.

The human intestinal epithelial cell lines AGS (stomach cell line) and Caco-2 (colon cell line) were used. The AGS cells in RPMI medium (Invitrogen) supplemented with 10% FCS (Invitrogen) and 50 μg/mL of vancomycin (Sandoz, Levallois Perret, France) and the Caco-2 cells were grown in Modified Eagle’s Medium (MEM) (Invitrogen, Fisher Scientific SAS, Illkirch Cedex, France) supplemented with 1% Minimum Essential Amino Acids (MEAA) (Invitrogen).

Lymphocytes were purified from peripheral blood from hemochromatosis patients by Ficoll gradient with therapeutic phlebotomy performed regularly at the EFS (French Blood Establishment, Aquitaine-Limousin) (No. EFS CPS 10.41 Convention). Blood was collected in bags (MSE 6500 L) (Macopharma, Tourcoing, France) in the presence of an anticoagulant (sodium citrate).

### Phylogenetic analysis

The *ggt* genes of *C. jejuni* strains 2003–198, 2002–200, 2004–304, 2003–383, 2004–438, 2002–608, 2002–646, 2007–741, 2003–795, 2003–1129, and 2003–1206 were amplified and sequenced using external and internal gene-specific primers (Table 2). The sequences obtained were translated into protein sequences using the EMBOSS software Transeq (http://www.ebi.ac.uk/Tools/st/emboss_transeq/). GGT sequences of 5 *C. jejuni* strains for which the genome is completely sequenced, were selected: *C. jejuni* strains 81116 [23], 81176 [24], M1 [25], ICDCCJ07001 [26] and *C. jejuni subsp. doylei* strain 269.97 (RefSeq NC_009707.1). GGTs of 8 published *H. pylori* strains were also selected: strains 26695 [27], J99 [28], HPAG1 [29], 908 [30], P12 [31], Shi470 [32], B38 [33] and B45 [34], as well as GGTs of *H. canis*, *H. bilis*, *H. trogontum, H. felis*[35], *H. salomonis*, *H. suis*[36], *H. bizzozeronii*[37], *H. cetorum, H. acinonychis*[38] and *H. mustelae*[39] strains. GGT sequences of 2 *Arcobacter* species (related to *Campylobacter* species) were added to our selection. Phylogenetic analysis of GGT amino-acid sequences was conducted in MEGA4 software using the Minimum Evolution (ME) method after alignment in the ClustalW2 software (http://www.ebi.ac.uk/Tools/msa/clustalw2/) and 1,000 repetitions of this analysis [40].

**Table 2:** Oligonucleotides used in this study

**Oligonucleotides (size)**	**Sequence (5′-3′)**
**F-GGT (23 bp)**	GGGTAAATAAGAAGTTAGAATTC
**R-GGT (21 bp)**	CTTGATAAAGGCGGAAATGCC
**F1GGT (20 bp)**	TGCTTTGGCTGTAGTGCATC
**R1GGT (20 bp)**	TGCTTACAGCATTGCCTTTG
**F2GGT (20 bp)**	TAGGCTTTTTGCGGTGGTAG
**R2GGT (20 bp)**	CAGGAGATCCTGTGCCTGTG
**F3GGT (21 bp)**	TTATCGCAAAGGAAGGTCCTG
**R3GGT (20 bp)**	AGCATCAGGACCTTCCTTTG

bp: base pairs.

Nucleotide and protein sequences for the 11 *C. jejuni* GGTs sequenced in the present study are available in Genebank (EMBL) under the following accession numbers: KF985027, KF991209, KF991210, KF991211, KF991212, KF991213, KF991214, KF991215, KF991216, KF991217, KF991218 for the strains 2002–200, 2002–608, 2002–646, 2003–198, 2003–383, 2003–1129, 2003–1206, 2004–304, 2004–438, 2007–741, and 2003–795 respectively.

### Enzyme assay for GGT activity

GGT enzymatic activity was determined by the spectrophotometric method described by Meister *et al.*[41]. The assay solution contained 20 mM of glycylglycine (Sigma Aldrich, Saint-Quentin Fallavier, France), 300 mM of L-γ-glutamyl-p-nitroanilide (Sigma Aldrich) and 60 mM of TRIS pH 8. The reaction mixture (5 μl of GGT in 200 μL of assay solution) was incubated at 37°C and analyzed by spectrophotometry at 405 nm to evaluate the release of p-nitroanilide (yellow compound). The activity of acivicin (Santa Cruz Biotechnology, Heidelberg, Germany), a pharmacological inhibitor of GGT, was also tested (10 μM) after a 2 h preincubation at 37°C as well as GGT heat inactivation at 70°C for 20 min

### *C. jejuni* GGT purification

A bacterial supernatant of *C. jejuni* 81116 was prepared by incubating the strain in 1 L of PBS overnight at 37°C with orbital shaking (150 rpm). After centrifugation at 10,000 rpm for 10 min, the supernatant was recovered. Solid ammonium sulfate (Sigma Aldrich) was added to the cell extract to 90% saturation, and the mixture was stirred at 4°C for 2 h. The precipitate was removed by centrifugation at 12,000 rpm for 30 min at 4°C and dissolved in 6 mL of dialysis buffer (TRIS 50 mM, NaCl 25 mM, pH 8). This solution was dialyzed twice against 1 L of the same buffer for 2 h and overnight at 4°C. The GGT activity was tested on 5 μL of the dialyzed solution according to the method of Meister *et al.* described above [41].

One mL of the dialyzed solution was applied to a MonoQ®HiTrap column (GE Healthcare, Aulnay sous Bois, France) equilibrated with 50 mM TRIS pH 8 (Buffer A). Elution was performed at a flow rate of 1 mL/min with a gradient of NaCl stepwise (0.08; 0.25; 0.35 and 1 M) in buffer A. 330 μl fractions were collected and tested for GGT activity as previously described. The active fractions were combined and SDS-PAGE was performed with 12% polyacrylamide running gel and 5% polyacrylamide stacking gel. After electrophoresis, the gel was stained with Coomassie blue.

The active fraction was then purified by a second ion exchange chromatography (MonoQ®HiTrap column, 1 mL, GE Healthcare). Elution was performed at a flow rate of 1 mL/min with a linear gradient of NaCl (0.08 to 0.5 M). The active fraction was not retained by the column and was eluted directly. SDS-PAGE and Coomassie blue staining were performed. The active fraction was precipitated with ammonium sulfate (90% saturation) at 4°C for 2 h. The precipitate was removed by centrifugation at 12,000 rpm for 30 min at 4°C and dissolved in 400 μL of dialysis buffer. This solution was dialyzed twice for 2 h each time against 500 mL of the same buffer at 4°C. SDS-PAGE and Coomassie blue staining were performed. The bands of interest were cut and analyzed by mass spectrometry at the Functional Genomics Platform of the University of Bordeaux. The protein concentration was determined spectrometrically using the Bradford method with bovine serum albumin (BSA) as a standard (Biorad, Marnes La Coquette, France). The GGT activity was again evaluated as described above.

### Mass spectrometry

Sample preparation: Each SDS-PAGE band was cut into 1 mm × 1 mm gel pieces. Gel pieces were destained in 25 mM ammonium bicarbonate, 50% acetonitrile (ACN) and shrunk in ACN for 10 min. After ACN removal, gel pieces were dried at room temperature. Proteins were first reduced in 10 mM dithiothreitol, 100 mM ammonium bicarbonate for 30 min at 56°C then alkylated in 100 mM iodoacetamide, 100 mM ammonium bicarbonate for 30 min at room temperature and shrunk in ACN for 10 min. After ACN removal, gel pieces were rehydrated with 100 mM ammonium bicarbonate for 10 min at room temperature. Before protein digestion, gel pieces were shrunk in ACN for 10 min and dried at room temperature. Proteins were digested by incubating each gel slice with 10 ng/μl of trypsin (T6567, Sigma-Aldrich) in 40 mM NH4HCO3, 10% ACN, rehydrated at 4°C for 10 min, and finally incubated overnight at 37°C. The resulting peptides were extracted from the gel in three steps: an initial incubation in 40 mM ammonium bicarbonate, 10% ACN for 15 min at room temperature and two incubations in 47.5% ACN, 5% formic acid for 15 min at room temperature. The three collected extractions were pooled with the initial digestion supernatant, dried in a SpeedVac, and resuspended in 25 μL of 0.1% formic acid before nanoLC-MS/MS analysis.

NanoLC-MS/MS analysis: Online nanoLC-MS/MS analyses were performed using an Ultimate 3000 system (Dionex, Amsterdam, The Netherlands) coupled to a nanospray LTQ Orbitrap XL mass spectrometer (Thermo Fisher Scientific, Bremen, Germany). Ten μL of each peptide extract were loaded on a 300 μm ID × mm PepMap C_18_ precolumn (LC Packings, Dionex, Sunnyvale, CA, USA) at a flow rate of 30 μL/min. After 5 min of desalting, peptides were separated on a 75 μm ID × 15 cm C_18_PepMap™ column (LC packings) with a 5-40% linear gradient of solvent B for 108 min (solvent A was 0.1 formic acid in 5% ACN and solvent B was 0.1% formic acid in 80% ACN). The separation flow rate was set at 200 nL/min. The mass spectrometer operated in a positive ion mode at a 1.8-kV needle voltage and a 27-V capillary voltage. Data were acquired in a data-dependent mode alternating an FTMS scan survey over the range m/z 300–1700 with the resolution set to a value of 60,000 at m/z 400 and 6 ion trap MS/MS scans with Collision Induced Dissociation (CID) as the activation mode. MS/MS spectra were acquired using a 3-m/z unit ion isolation window and normalized collision energy of 35. Mono-charged ions and unassigned charge-state ions were rejected from fragmentation. Dynamic exclusion duration was set to 30 sec.

Database search and results processing: Mascot and Sequest algorithms through Proteome Discoverer 1.4 Software (Thermo Fisher Scientific) were used for protein identification in batch mode by searching against a *C. jejuni* UniProt database (44,546 entries). Two missed enzyme cleavages were allowed. Mass tolerances in MS and MS/MS were set to 10 ppm and 0.8 Da. Oxidation of methionine and carbamidomethylation on cysteine were searched as variable modifications. Peptide validation was performed using Percolator algorithm [42] and only “high confidence” peptides were retained corresponding to a 1% false positive rate at peptide level.

### *C. jejuni* GGT activity on epithelial cells

Cell proliferation: Epithelial cells were cultured in 96-well plates for 48 h. The effect of purified GGT on these lines was evaluated at different concentrations (2.5 to 320 ng/mL) after 24 h of treatment with the MTT Formazan kit (Sigma-Aldrich). GGTs, either preincubated for 2 h in the presence of acivicin or previously heat-inactivated (70°C, 20 min), were also tested.

Cell apoptosis: AGS cells were cultured in 24-well plates for 48 h. The pro-apoptotic effect of GGT (10 ng/mL) with and without acivicin after 24 h of treatment was evaluated using the Nicoletti method [43]. Staurosporine (10 μM) (Sigma-Aldrich), a pro-apoptotic compound, was used as a positive control. Briefly, adherent cells were trypsinized, washed and centrifuged for 5 min at 3,000 rpm. The cell pellet was resuspended in 100 μL of Nicoletti buffer (0.1% sodium citrate, 0.1% Triton X-100, 50 mg/L of propidium iodide in distilled water). The FACS CantoII cytometer (BD Biosciences, Le Pont de Claix, France) was used for data acquisition.

### *C. jejuni* GGT activity on lymphocytes

Lymphocytes were cultured in 24-well plates (1.10^6^ cells/well) for 4 days at 37°C in a 5% CO_2_ atmosphere in RPMI supplemented with 10% FCS and 50 μg/mL of vancomycin in the presence of purified GGT (10 ng/mL) with or without acivicin (10 μM), and heat-inactivated (70°C, 20 min) or not. The proliferative capacity of lymphocytes was monitored using phytohemagglutinin (PHA, 1 μg/mL) (Sigma Aldrich) and interleukin 2 (IL 2, 1000 U/mL) (Chiron, Surennes, France) [19]. Lymphocyte proliferation was measured by BrdU incorporation (5 bromo 2′ deoxyuridine) (BD Biosciences) with flow cytometry (FACS CantoII) using a method previously validated in the laboratory [19]. This incorporation was revealed using an anti-BrdU antibody coupled to the fluorochrome fluorescein isothiocyanate (FITC) (BD Biosciences). Stimulation of this fluorochrome by the laser cytometer allowed an analysis of the cells based on their size and density and a record of the proportion of those that have been specifically recognized by the anti-BrdU antibody. The results were analyzed using CellQuest (BD Biosciences) software and were used to determine the percentage of proliferated cells.

During the same experiment, the percentage of cells undergoing apoptosis and the cell cycle were both evaluated by the Nicolleti method. Lymphocytes were centrifuged for 10 min at 1,500 rpm and resuspended in 100 μl of Nicoletti buffer.

### Statistical analysis

Statistical analyses were performed using the Mann–Whitney test (SPSS Version 16 software). This is a nonparametric test which is used to test the equality of distribution of two independent sets of values to be compared (p <0.05).

## Abbreviations

GGT: Gamma-glutamyl transpeptidase; PI: Propidium iodure; Ly: Lymphocytes.

## Competing interests

The authors declare that they have no competing interests.

## Authors’ contributions

PF, VPey, AHDLG and JWD performed the experiments. PF, PL, FM, JWD wrote the manuscript. PL, PF and MC designed the experiments. VPitard, MB supervised some of the data analysis. All authors read and approved the final manuscript.

## Authors’ information

FM and PL are the head and co-head of the National Reference Center for Campylobacters and Helicobacters, respectively.

## Supplementary Material

Additional file 1: Figure S1 Tree based on amino-acid sequences of different bacterial GGTs. The evolutionary history was inferred using the method of “Minimum Evolution” and the evolutionary distances were calculated using the matrix method of Dayhoff. The scale indicates the amino-acid substitutions. The numbers next to the branches indicate the robustness of the separation of the branches in the tree obtained (>70%, analysis repeated 1,000 times). Click here for file

Additional file 2: Figure S2 Example of cell cycle evaluation by flow cytometry (Nicoletti assay). (1) Selection of the population of interest (P2) by freeing aggregated cells that give false cells in the G2/M phase and taking into consideration the width of the emission wavelength of phycoerythrin (PE-W) according to the peak area (PE-A). From P2, Graphs (2) control lymphocytes, (3) Lymphocytes with *C. jejuni* GGT (10 ng/mL) and (4) lymphocytes with *C. jejuni* GGT (10 ng/mL) preincubated with acivicin (10 μM) allow a distinction between G0/1 (P3), S (P4) and G2/M (P5) lymphocytes. Click here for file
