# Supplementary figures and images for: Role of Campylobacter jejuni gamma-glutamyl transpeptidase on epithelial cell apoptosis and lymphocyte proliferation
Source: Gut Pathog. 2014 Jun 12;6:20. doi: 10.1186/1757-4749-6-20 (PMC4080688; doi:10.1186/1757-4749-6-20)

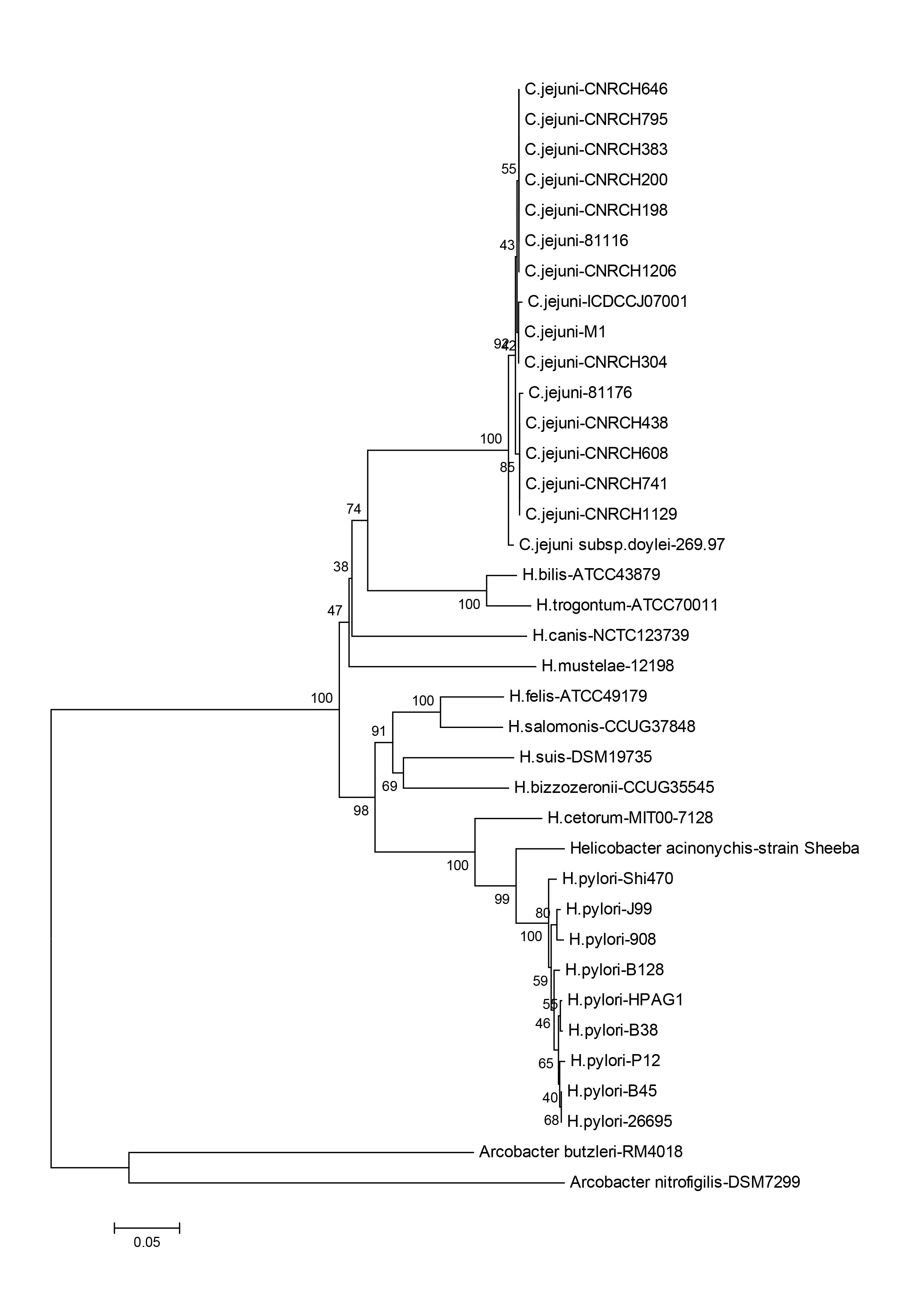

Supplement: Additional file 1: Figure S1 — Tree based on amino-acid sequences of different bacterial GGTs. The evolutionary history was inferred using the method of “Minimum Evolution” and the evolutionary distances were calculated using the matrix method of Dayhoff. The scale indicates the amino-acid substitutions. The numbers next to the branches indicate the robustness of the separation of the branches in the tree obtained (>70%, analysis repeated 1,000 times). [file 1757-4749-6-20-S1.jpeg]

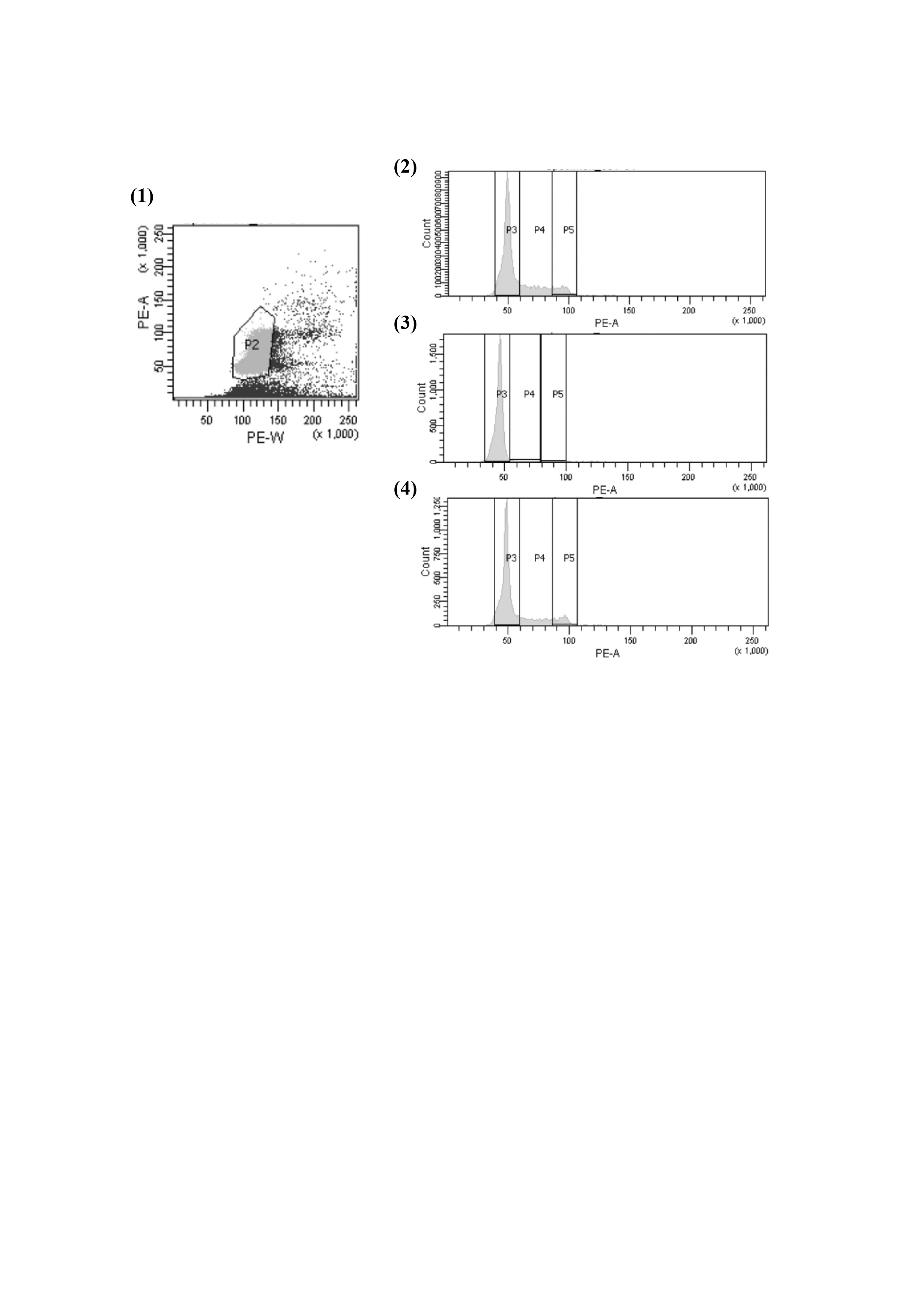

Supplement: Additional file 2: Figure S2 — Example of cell cycle evaluation by flow cytometry (Nicoletti assay). (1) Selection of the population of interest (P2) by freeing aggregated cells that give false cells in the G2/M phase and taking into consideration the width of the emission wavelength of phycoerythrin (PE-W) according to the peak area (PE-A). From P2, Graphs (2) control lymphocytes, (3) Lymphocytes with C. jejuni GGT (10 ng/mL) and (4) lymphocytes with C. jejuni GGT (10 ng/mL) preincubated with acivicin (10 μM) allow a distinction between G0/1 (P3), S (P4) and G2/M (P5) lymphocytes. [file 1757-4749-6-20-S2.jpeg]
